# Research Lumbar Punctures among African Americans and Caucasians: Perception Predicts Experience

**DOI:** 10.3389/fnagi.2016.00296

**Published:** 2016-12-02

**Authors:** Jennifer C. Howell, Monica W. Parker, Kelly D. Watts, Alexander Kollhoff, Dobromira Z. Tsvetkova, William T. Hu

**Affiliations:** ^1^Department of Neurology, Emory University School of MedicineAtlanta, GA, USA; ^2^Center for Neurodegenerative Diseases Research, Emory University School of MedicineAtlanta, GA, USA; ^3^Alzheimer’s Disease Research Center, Emory University School of MedicineAtlanta, GA, USA

**Keywords:** Alzheimer’s disease, cerebrospinal fluid, health disparity

## Abstract

African Americans are under-represented in Alzheimer’s disease (AD)-related biomarker studies, and it has been speculated that mistrust plays a major factor in the recruitment of African Americans for studies involving invasive procedures such as the lumbar puncture (LP). We set out to determine factors associated with non-participation in a biomarker study aiming to explore cerebrospinal fluid (CSF) AD biomarker differences between older African Americans and Caucasians. We also surveyed participants’ procedure-related perception (a standard medical procedure vs. a frightening invasive procedure) and reluctance, as well as the rate and type of post-procedure discomfort and complications. Among 288 subjects approached for study participation, 145 (50.3%) refused participation with concerns over LP being the most commonly reported reason. Relatively more African Americans than Caucasians reported concerns over LP as the main reason for non-participation (46% vs. 25%, *p* = 0.03), but more African Americans also did not provide a specific reason for non-participation. Among those who completed study participation (including the LP), African Americans and Caucasians were similar in pre-LP perceptions and reluctance, as well as post-LP rates of discomfort or complication. Perceiving LP as a frightening invasive procedure, not race, is associated with increased likelihood of post-LP discomfort or complication (RR 6.2, 95% confidence interval 1.1–37.0). Our results indicate that LP is a well perceived procedure in a cohort of African American and Caucasian research participants, and is associated with few serious complications. The pre-procedure perception that the LP is a frightening invasive procedure significantly increases the risk of self-reported discomfort of complications, and African Americans may be more likely to turn down study participation because of the LP. Future studies will need to address factors associated with negative LP perceptions to further assure participants and reduce complication rates.

## Introduction

The ante-mortem diagnosis of Alzheimer’s disease (AD) in people with very mild symptoms can be enhanced by incorporating etiologic biomarkers into the diagnostic algorithm. Large studies incorporating cerebrospinal fluid (CSF) biomarkers have characterized findings from mostly Caucasian cohorts, reflecting the general trend of lower African American participation in AD-related research (Li et al., 2007; Wahrle et al., 2007; Aisen et al., 2010; Moghekar et al., 2013; Shin and Doraiswamy, 2016). We recently showed that CSF AD biomarker profiles differentially tracked cognitive decline according to race, and findings from cohorts recruited at specialty centers may not be directly applicable to the broader multi-racial population especially given the phenotypic differences in AD between Caucasians and minority populations (Schupf et al., 2008; Lee et al., 2012). With international studies designed after the North American AD Neuro-imaging Initiative (ADNI) to address the relationship between cognitive decline, biomarkers and racial background, there is an urgent need to apply modern biomarkers to the aging minority population in the US.

There are several potential barriers to African American participation in AD-related research (Shin and Doraiswamy, 2016). Community-dwelling African Americans previously reported mistrust of researchers, institutions/healthcare; integrity of research studies; and fear of the unknown (procedures including lumbar puncture (LP) and medications) to influence their willingness to volunteer for AD biomarker research (Williams et al., 2010). Historical events surrounding race relations, such as the Tuskegee Study of Untreated Syphilis (TSUS) and the Henrietta Lacks case, were previously identified to limit research participation (Shavers et al., 2000; Underwood et al., 2013), but their direct impact on AD-related biomarker research remains unknown. Furthermore, few studies have reported on the rate of LP complications in African Americans compared to Caucasians, and none has directly examined the relationship between pre-LP perception and the LP experience in African Americans. As part of the broader effort to define CSF, MRI and molecular imaging biomarker profiles of cognitive decline in this group, we assessed the relationship between inclusion of LP in the study and participation rate, and the connection between pre-LP views and post-LP complications according to race.

## Materials and Methods

### Participants

Potential participants between the ages of 55 and 90 were recruited from individuals attending Emory-sponsored community outreach events in the greater Atlanta area, undergoing memory evaluation at the Emory Cognitive Neurology Clinic, or already participating in observational studies at the Emory University from February 2014 to September 2015. Recruitment occurred from February 2014 to September 2015. The study was approved by Emory University Institutional Review Board, and written informed consents were obtained from all participants or their legal representatives when appropriate.

### Study Design

Two hundred and eighty-eight potential participants were initially contacted for the biomarker study by phone, regular mail, e-mail, or in-person discussion. Study staff familiar with the biomarker study procedures (neuropsychological testing, phlebotomy, LP, MRI, molecular imaging) explained all aspects of the study to potential participants. For the LP, study staff discussed the purpose, study design, process, analgesia, potential complications and their treatments with each participant and allowed sufficient time for all questions to be answered. If a subject declined participation in the study, he or she was asked for the main refusal reason in open-ended question format. The reason was recorded in a free text form. After the necessary recruitment goal for the biomarker study was reached, reasons for refusal were reviewed and categorized as follows: not interested, too busy, too much distance to travel, concerns over LP, claustrophobia, general anxiety over testing (other than LP or MRI), personal health reasons, lack of family support to participate in study, distrust associated with the TSUS, and no reason provided.

All participants undergoing the LP were asked to complete the AD Center Patient LP Experience Survey (with permission from Dr. John Morris, Washington University^1^). There are two parts to the survey. The pre-LP portion included questions on general demographic information: clinical diagnosis, age, gender, race, ethnicity (Hispanic or non-Hispanic) and education (high school or less, associate degree, bachelor’s degree, master’s degree or higher); past history of headache and pain disorder; perception of LP (“standard medical procedure” or a “frightening, invasive procedure”); and attitudes toward undergoing an LP (“calm, no problems”, “somewhat reluctant” and “very reluctant”). Prior to the procedure, informed consent involving the clinician performing the LP with a focus on potential adverse events and ways to reduce their risks (atraumatic needle, iodine or chlorhexidine use for insertion site antisepsis, brief rest after the procedure, increased caffeine and hydration and avoiding heavy lifting or exertion) took place in a private room with the subject and, if necessary, a legal representative. The post-LP portion was completed via a telephone call or email from study staff 7 days after the LP to inquire about any complications including needle insertion site pain, back stiffness, non-specific headache and post-lumbar puncture headache (PLPH). Subjects who reported any of these or other symptoms were asked to elaborate on the quality of the symptoms, with specific questions on intensity, duration and treatment (if any) for PLPH. Eighty-six (67%) participants completed the LP survey. Compared to those who declined the survey portion of the study, those who completed the surveys were more likely to be African Americans (56% vs. 26%, *p* < 0.001) and have master’s degree or higher education (48% vs. 26%, *p* < 0.02), but were otherwise similar in age and gender (Table 1). How each participant came into contact with the study (new patients undergoing evaluation, *n* = 13; established patients volunteering for the study, *n* = 15; non-patients volunteering for the study, *n* = 56) was also recorded.

**Table 1:** **Demographic features of lumbar puncture (LP) participants who did or did not complete the pre- and post-LP surveys**.

	Completed survey (*n* = 86)	Did not complete survey (*n* = 42)	*p*
African American (%)	48 (55%)	11 (26%)	0.001
Male (%)	40 (46%)	18 (43%)	0.421
Diagnosis			0.575
Normal cognition	38 (44%)	15 (36%)	
MCI	31 (36%)	19 (45%)	
AD dementia	17 (20%)	8 (19%)	
Age			0.734
55–64	20 (23%)	10 (24%)	
65–74	40 (47%)	23 (55%)	
75–84	24 (28%)	8 (19%)	
>84	2 (2%)	1 (2%)	
Education			0.162
High School or less	13 (15%)	11 (26%)	
Associate’s degree	10 (12%)	5 (12%)	
Bachelor’s degree	22 (25%)	15 (36%)	
Master’s degree or higher	41 (48%)	11 (26%)	

### Statistical Analyses

All statistical analyses were performed using SPSS 22 (IBM SPSS, Armonk, NY, USA). Basic demographics were compared using Chi-squared tests according to race, perception of LP and complication type (any or specific complications). Factors identified to be associated with each outcome at the univariate level with *p* < 0.20 were entered into multi-variate binary logistic regression model in a backward likelihood ratio approach until *p* < 0.10.

## Results

**Figure 1:**
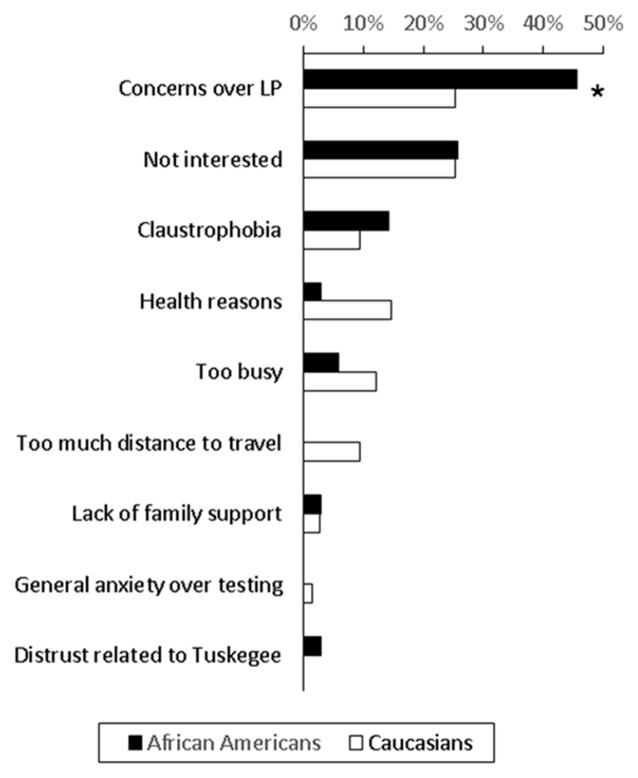
**Reasons for non-participation among African Americans and Caucasians who declined to undergo a biomarker study involving lumbar puncture (LP) and MRI**. 59% of African Americans and 87% of Caucasians who did not participate in the biomarker study provided the main reason for non-participation, and these reasons are shown as percentage of all reasons (*p = 0.03).

One hundred and twenty-eight (44%) of contacted subjects consented to study participation, 145 (50.3%) declined participation and 15 (5%) subjects were still considering participation. The most common refusal reason was concerns over the LP, accounting for 22.1% of Caucasian and 27.1% of African American adults who declined participation (Figure 1). When we only examined subjects who provided a clear reason for non-participation, African Americans were more likely to express concerns over LP than Caucasians (45.7% vs. 25.3%, *p* = 0.03). Only one subject (1.7% of African Americans contacted) brought up concerns stemming from knowledge of TSUS.

Eighty-six of the biomarker study participants completed the LP survey, including 48 African Americans (82% of those who underwent LP) and 38 Caucasians (56% of those who underwent LP). African American and Caucasian respondents were similar in age, gender, education, cognitive status, contact with study and past experiences with headaches, chronic pain disorder and LPs (Table 2). The two racial groups were also similar in terms of the LP needle used (atraumatic vs. standard cutting), pre-procedure LP perception (standard vs. frightening invasive) and attitudes (calm vs. reluctant, Table 3). Over one quarter of participants (27.9%) reported some physical discomfort or complication during or following the procedure, including four participants who had two types of symptoms. Needle insertion site pain was the most common complaint (*n* = 13, 15.1%), followed by headache of any kind (*n* = 11, 13.1%), back pain/stiffness (*n* = 4, 4.7%), and non-specific leg tingling (*n* = 1, 1%). Seven cases of headaches were postural in nature consistent with PLPH (8%). All seven subjects described the PLPH to be mild in intensity, and most (6/7) experienced the onset of symptoms 2–24 h after the LP. All cases of suspected PLPH were self-limited without need for blood patching, and four cases lasted more than 24 h in duration (range: 2–4 days).

**Table 2:** **Demographic information for LP survey respondents**.

	African American (*n* = 48)	Caucasian (*n* = 38)	*p*
Male	23 (48%)	17 (45%)	0.772
Age			0.214
55–64	14 (29%)	6 (16%)	
65–74	21 (44%)	19 (50%)	
75–84	12 (25%)	12 (32%)	
>84	1 (2%)	1 (3%)	
Diagnosis			0.839
Normal cognition	20 (42%)	18 (47%)	
MCI	19 (40%)	12 (32%)	
AD	9 (19%)	8 (21%)	
Education			0.584
High school or less	9 (19%)	4 (11%)	
Associate’s degree	5 (10%)	5 (13%)	
Bachelor’s degree	11 (23%)	11 (29%)	
Master’s degree or higher	23 (48%)	18 (47%)	
Contact with study			0.476
New patients undergoing evaluation	6 (12%)	7 (18%)	
Established patients volunteering	7 (15%)	8 (21%)	
Non-patient volunteers	35 (73%)	23 (61%)	
Past headaches			0.392
None	43 (90%)	32	
Mild	4 (8%)	4	
Chronic	1 (2%)	2	
Past pain disorders			0.221
None	40 (83%)	36 (95%)	
Mild	4 (8%)	0 (0%)	
Chronic	4 (8%)	2 (5%)	
Prior LP experience	9 (19%)	5 (13%)	0.465

**Table 3:** **Technical factors and discomfort and complications associated with LPs among survey respondents**.

	African Americans (*n* = 48)	Caucasians (*n* = 38)	*p*
Atraumatic needle was used (%)	40 (83%)	34 (89%)	0.537
Views LP as a frightening invasive procedure	3 (6%)	3 (8%)	1.000
Reluctant or somewhat reluctant (%)	5 (10%)	8 (21%)	0.171
Needle site injection pain (%)	7 (15%)	6 (16%)	0.877
Back pain/stiffness (%)	3 (6%)	1 (3%)	0.627
Any headache (%)	7 (15%)	4 (10%)	0.748
PLPH (%)			1.000
Mild	4 (8%)	3 (8%)	
Moderate to severe	0	0	

**Figure 2:**
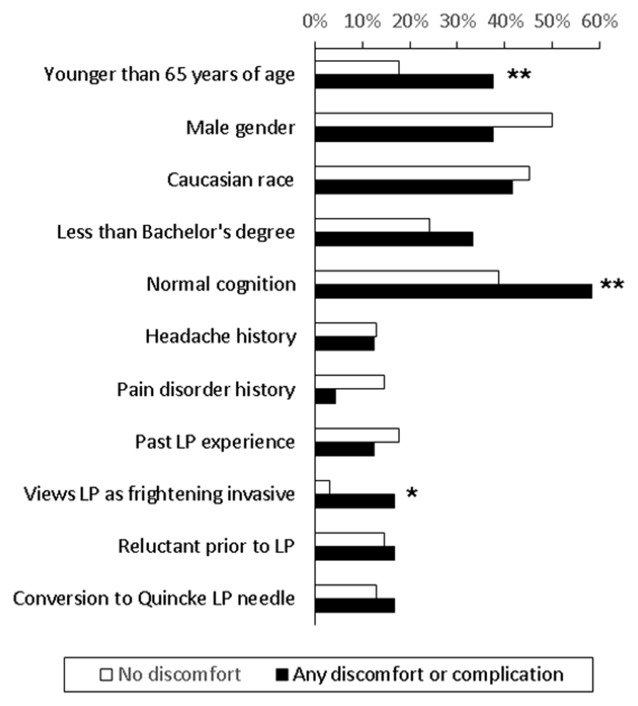
**Comparison of subjects who did (*n* = 24) and did not (*n* = 62) report discomfort or complications**. *More common among those who reported discomfort or complication, *p* < 0.05. **More common among those who reported discomfort or complication, 0.10 > *p* ≥ 0.05.

In examining factors associated with any discomfort or complication, univariate analysis showed that perceiving LP as frightening and invasive was more common in those reporting discomfort or complication than those who did not report any (16.7% vs. 3.2%, *p* = 0.049). There was also a trend that normal cognition (*p* = 0.100) and age younger than 65 (*p* = 0.052) were associated with any discomfort or complication (Figure 2). Other factors, including how each participant initially came into contact with the study, did not influence the rate of discomfort or complications. Multi-variate logistic regression analysis including these three variables showed that negative LP perception was the only factor associated with increased risk (6.2, 95% confidence interval 1.1–37.0) of any self-reported discomfort or complications. When each discomfort or complication was analyzed individually, needle insertion site pain was associated with negative LP perception (3/6 vs. 10/68, *p* = 0.045) and age younger than 65 (7/20 vs. 6/66, *p* = 0.005), back pain/stiffness and PLPH were associated with less than bachelor’s degree in education (3/23 vs. 1/63, *p* = 0.057; 4/23 vs. 3/63, *p* = 0.079), and any headache was associated with normal cognition (8/38 vs. 3/48, *p* = 0.054). The relationship between younger age and needle insertion site pain (*p* = 0.02) persisted in the multi-variate regression analysis. In this cohort, any history of headache or pain disorder was not associated with higher complication rates.

## Discussion

The LP represents a widely available and cost-effective method to obtain etiologic AD biomarkers. Among AD researchers, there is a common belief that the general public is resistant to undergoing diagnostic and research LPs alike. Here we confirm that concern over LP was a common reason for older Americans to decline participation in an AD biomarker study, and this concern is potentially greater among older African Americans than Caucasians. However, among those who consented to undergo LPs, race was not associated with worse perception, greater reluctance, or higher rates of complication. Instead, a negative pre-LP perception of the procedure predicted discomfort and complications for both races. Thus, perception of LP may not only influence participation rates in AD biomarker studies but also rates of complications following study procedures.

It is not known if concern over LP among those who declined study participation was associated with unfamiliarity with the procedure, negative personal experience with LP, mistrust in the medical system, or other factors. Proportionally more African Americans referenced LP concerns as a reason for non-participation than Caucasians, and our anecdotal experience suggests that most fears are centered around pain, needles and paralysis rather than death or meningitis (Thakur et al., 2015). While TSUS was only directly referenced once as the primary reason for non-participation, it is possible that more African Americans reported personal LP fear or declined to provide a specific reason for non-participation rather than referencing TSUS outright. However, the Tuskegee Legacy Project has found no support for the notion that African Americans were more reluctant to participate in research than Caucasians because of greater TSUS knowledge (Katz et al., 2006, 2008). Thus, it is more likely that fear of personal injury and complications are of greater cause for concern than the legacy of TSUS. Having had a prior LP did not appear to influence the pre-procedure perception or attitudes in our cohort, but subjects with negative experiences may have declined participation to bias this finding. Other factors may also have stronger influence on African Americans’ willingness to participate in research than knowledge of TSUS, including knowing someone who has undergone LPs, personal or family exposure to neurological disorders, trust in research (Kennedy et al., 2007; Danner et al., 2008; Williams et al., 2010), altruism (Williams et al., 2010), and general health status. A detailed examination of these more personalized factors among potential research volunteers may yield greater minority participation than educational or advocacy activities surrounding TSUS.

We found a relatively high rate of post-LP discomfort and complications in our cohort. We are hesitant to interpret this figure (28%) solely as complication or adverse event rate, as some symptoms such as mild back pain at the needle insertion site were previously considered expected events rather than adverse events (Peskind et al., 2005). A large multi-center European study (BIOMARKAPD) with 3456 subjects reported a similar overall complication rate of 30.8%, including 17% reporting any back pain and 19% reporting any headaches (PLPH or not) following the LP (Duits et al., 2016). Both our study and BIOMARKAPD had higher PLPH rates (8% and 9%) than two previously published studies, but the differences most probably resulted from severity-based definitions of PLPH (Peskind et al., 2005) and reliance on patient self-reporting to document complications (Zetterberg et al., 2010). Using alternate criteria, our cohort had very low rates of adverse event: zero case of PLPH of moderate severity, three cases of transient backache beyond needle site insertion pain, and one case of transient lower extremity paresthesia in a patient with prior radiculopathy. For standardized reporting and quality control, professional associations, research sponsors and regulators should better define the need to report duration and intensity of symptoms by different investigators, even if these symptoms are short of adverse events requiring intervention.

How negative LP perceptions can mechanistically lead to higher rates of discomfort and complications is of interest to prevent these unpleasant, if not adverse, events. BIOMARKAPD investigators found similar relationship between anxiety and outcomes, and biopsy studies also showed perceived pain to relate to the pre-procedure anticipated level of pain (Soo et al., 2014; Humbyrd et al., 2016). Potential mechanisms to account for these associations include impaired inflammatory response to injury in chronic (Kiecolt-Glaser et al., 1995) or acute stress (Marucha et al., 1998), aversive conditioning related to stress (Robinson et al., 2013), neuro-endocrine alterations (Catania et al., 2009) and shared genetic susceptibility (Zhou et al., 2008). It remains to be prospectively determined if technical factors designed to reduce complications (Hilzenrat et al., 2006; Patout et al., 2015) will impact state anxiety and individuals’ LP experience. Future studies aimed at improving pre-LP perception to enhance the experience of patients and study participants should examine other factors which impact anxiety.

To the best of our knowledge, this is the first work of its kind to focus on differences in African American and Caucasian recruitment into a modern AD biomarker study. We were able to examine real, rather than theoretical barriers in recruiting African Americans into a study involving a single LP, and did not find significant concerns related to TSUS. At the same time, the cohort we contacted for the biomarker study may not be representative of the broader community as many subjects are familiar with our work related to CSF biomarkers and health services research (Hu et al., 2015; Howell et al., 2016). We were not able to solicit a specific reason for a significant proportion of African Americans who declined research participation, and this itself may represent some form of mistrust. Similarly, we did not explore any underlying motivation for research participation (e.g., altruism, family history, personal concern) which may influence pre-procedure attitudes, although the rate of discomfort or complication was not influenced by whether the participant was seeking or receiving routine clinical care at our institution. Finally, the cohort we approached may lack the power to detect differences between the racial groups (e.g., income), and we did not assess other factors which may influence research participation such as personal exposure to AD (first degree relative, friend, etc.), prior experience in research studies, and trust of the modern medical system. So while African Americans were perhaps more likely to turn down research participation due to concerns over LP than Caucasians in our study, more work is necessary to determine whether other background factors influence research participation in a community cohort without direct exposure to aging research (Howell et al., 2016). At the same time, the low rates of serious adverse events and the lack of association between race and LP experience are reassuring. We propose that safety data from this study, proactive engagement in the community and timely dissemination of study findings relevant to participant health have the potential of greatly enhancing African American research participation, such that we and others can more readily employ modern AD biomarkers to address phenotypic differences in AD between the races (Shin and Doraiswamy, 2016).

## Author Contributions

JCH is responsible for design/conception of the study; acquisition and analysis of the data; and drafting of the manuscript. MWP is responsible for design/conceptualization of the study; acquisition, analysis and interpretation of the data; and critical revision of the manuscript for important intellectual content. KDW is responsible for design/conception of the study; analysis and interpretation of the data; and critical revision of the manuscript for important intellectual content. AK is responsible for acquisition and analysis of the data; and critical revision of the manuscript for important intellectual content. DZT is responsible for acquisition, analysis and interpretation of the data; and critical revision of the manuscript for important intellectual content. WTH is responsible for the design/conception of the study; acquisition, analysis and interpretation of data; and drafting of the manuscript. All authors have approved the final version of the manuscript, and all authors agree to be accountable for all aspects of the work.

## Funding

This work was supported by NIH AG43885, AG42856 and AG25688.

## Conflict of Interest Statement

WTH has a patent on cerebrospinal fluid-based diagnosis of Alzheimer’s disease and frontotemopral lobar degeneration through Emory University; consults for AARP, Inc. and Locks Law Firm; has received research support from Avid Pharmaceuticals; and has received travel support from Eli Lilly and Hoffman LaRoche. Other authors declare that the research was conducted in the absence of any commercial or financial relationships that could be construed as a potential conflict of interest.

## Footnotes

^1^https://www.alz.org/research/funding/partnerships/global_biomarkers/US_LP_Survey.pdf
